# Exploring Factors Affecting Health-Related Quality of Life in Coronary Artery Disease Patients

**DOI:** 10.3390/medicina61050824

**Published:** 2025-04-29

**Authors:** Geetha Kandasamy, Thangamani Subramani, Mona Almanasef, Khalid Orayj, Eman Shorog, Asma M. Alshahrani, Tahani S. Alanazi, Anusha Majeed

**Affiliations:** 1 Department of Clinical Pharmacy, College of Pharmacy, King Khalid University, Abha 62521, Saudi Arabia; glakshmi@kku.edu.sa (G.K.); malmanasaef@kku.edu.sa (M.A.); korayg@kku.edu.sa (K.O.); eshorog@kku.edu.sa (E.S.);; 2 Department of Pharmacy Practice, Grace College of Pharmacy, Palakkad 678004, India; 3 Department of Clinical Pharmacy, College of Pharmacy, Shaqra University, Dawadimi 11911, Saudi Arabia

**Keywords:** coronary artery disease, quality of Life, lifestyle factors, EQ-5D-3L

## Abstract

*Background and Objectives:* Coronary artery disease (CAD) significantly impacts health-related quality of life (HRQoL), with lifestyle factors and comorbidities influencing various dimensions of well-being. This study aimed to assess HRQoL and its association with sociodemographic and lifestyle factors in CAD patients. *Materials and Methods:* A cross-sectional study was conducted at the Rajiv Gandhi Co-operative Multispecialty Hospital, South India, from July 2022 to April 2023, where lifestyle factors were assessed, and HRQoL was measured using the EQ-5D-3L. The differences and associations of sociodemographic and lifestyle factors with HRQoL were analyzed using the chi-square test and multivariate regression. *Results:* A total of 212 CAD patients were included in this study. Female gender and comorbid disease were more likely to be associated with reported problems in mobility (89.7%, 78.8%) and anxiety/depression (97.4%, 92.7%) (*p* < 0.05). Factors such as age ≥ 50 years, family history of CAD, current smoking, comorbid disease, and a moderate- to high-risk diet significantly influenced anxiety/depression (*p* < 0.05). Patients with comorbid disease and moderate- to high-risk dietary intake were significantly associated with all five dimensions (*p* < 0.05). Gender, educational level, alcohol intake, and sleep duration did not show a significant association with all dimensions (*p* > 0.05). *Conclusions:* This study found that CAD patients undergoing treatment for secondary prevention exhibited inadequate HRQoL, particularly in terms of mental health. Factors such as comorbid disease and moderate- to high-risk dietary intake were significantly associated with reduced HRQoL. Older age, family history of CAD, current smoking habit, comorbid disease, and a moderate- to high-risk diet were significantly associated with anxiety/depression.

## 1. Introduction

Coronary artery disease (CAD) is the most prevalent form of cardiovascular disease (CVD) and is one of the primary causes of death, illness, and economic strain globally [1,2,3]. Recent research has shown that individuals with CAD have a significantly higher likelihood of experiencing cognitive impairment compared with healthy individuals of the same age [4,5,6]. Furthermore, the rates of anxiety and depression among heart patients are four times higher (15–20%) than those observed in the general population (5%), which negatively impacts health-related quality of life (HRQoL) and leads to increased morbidity and mortality [7,8,9]. The hallmarks of coronary heart disease (CHD) include the accumulation of cholesterol in the major coronary arteries, which can lead to angina, dyspnoea, fatigue, and myocardial infarction [10].

CHD is one of the most prevalent diseases, constituting a significant global public health concern. It is the leading cause of premature deaths from CVD, alongside stroke [11]. Despite a reduction in the CHD mortality rate over the past 20 years, low- and middle-income countries have experienced an increase in the number of life years lost to premature CHD-related deaths [12]. The World Health Organization (WHO) defines quality of life (QoL) as “an individual’s way of perceiving their life position within the cultural context and value system in which they live, and in relation to the tasks, expectations, and standards determined by environmental conditions” [13,14]. In the context of medicine, studying quality of life involves identifying issues arising from an illness and its treatment, linking these to human activity in physical, mental, and social domains and characterizing a patient’s perceptions of their subjective well-being and health [15,16].

The QoL of individuals with CAD is affected by various factors. Physical symptoms can impair functioning in familial and social contexts, often leading to social isolation [17]. A decline in physical ability can reduce independence, increasing reliance on others. Additionally, mobility limitations due to fatigue, dyspnea, muscle weakness, and poor balance can disrupt daily activities [18]. HRQoL is a multidimensional construct that encompasses an individual’s capacity to engage in daily activities (i.e., functioning), their overall life perspective (i.e., well-being), and their subjective assessment of health condition management. In recent years, HRQoL has emerged as a critical patient-reported outcome measure, providing valuable insights for patient-centered care, informing clinical decision-making, and influencing health policy and reimbursement frameworks [19]. Its integration into healthcare research and practice enhances the understanding of patient experiences and supports the development of targeted interventions to optimize health outcomes [20]. Furthermore, HRQoL has been identified as a significant predictor of mortality, recurrent coronary events, and long-term health prognosis [21]. Emerging evidence suggests that subjective well-being (SWB) should be considered when assessing overall well-being, as it encompasses a broad spectrum of psychological and evaluative dimensions, including emotional responses, domain-specific satisfaction, and global life satisfaction [22]. Given these insights, SWB may represent an additional critical indicator of HRQoL in patients with CHD, providing a more comprehensive assessment of their health status and QoL [23]. The aim of this study was to assess the HRQoL of patients with CAD and their associated lifestyle factors.

## 2. Materials and Methods

### 2.1. Study Design and Setting

A cross-sectional study was conducted at the Rajiv Gandhi Co-operative Multispecialty Hospital in Palakkad, Kerala, South India over a period of 8 months, from July 2022 to April 2023.

### 2.2. Sample Size and Study Criteria

Over an 8-month period, approximately 14 to 15 patients per week visited the cardiology outpatient department, resulting in a total sample of 470 patients. The sample size was determined using the Raosoft online calculator with a 5% margin of error, a 95% confidence level, and a 50% response distribution, yielding a minimum required sample size of 212, which was the total number of patients included in this study. The eligibility criteria required patients to be 18 years or older and to be diagnosed with CAD. Both male and female outpatients were considered, regardless of the presence of comorbidities. The exclusion criteria encompassed patients with severe non-cardiovascular diseases or conditions that could significantly impact life expectancy (e.g., cancer, drug abuse), those with impaired cognitive function, and individuals unwilling to provide informed consent.

### 2.3. Study Procedure and Data Collection

This study’s population consisted of patients diagnosed with CAD. Signed informed consent was obtained from each patient prior to this study. A predesigned patient data collection form was used to gather the required information. The data collection was divided into three sections: Section 1 included sociodemographic details, such as gender, age, education, occupation, marital status, family history of CAD, type of CAD, and comorbidities. Section 2 included lifestyle factors (exercise, smoking, alcohol consumption, sleep, and diet). The diet section comprised 10 questions assessing dietary habits, including frequency of fried food consumption; daily servings of bread, rice, potatoes, or other starchy foods; daily servings of sweet foods (e.g., cakes, biscuits, chocolate); daily teaspoons of sugar added to hot drinks or food; frequency of fish consumption; daily fruit intake; daily servings of vegetables (excluding potatoes); daily coffee consumption; average soft drink consumption; and daily water intake. These questions were adopted from a cardiovascular risk assessment questionnaire [24], with scores ranging from −10 to 10. Based on the total score, dietary habits were categorized as follows: Low risk: scores from −19 to 6; Medium risk: scores from 7 to 13; and High risk: scores of 14 and above. Section 3 included the assessment of QoL using the EQ-5D-3L questionnaire, which is a widely used tool for evaluating HRQoL. The questionnaire was translated into the local language, Malayalam, with the help of experts. To check reliability, internal consistency was measured using Cronbach’s alpha. The Cronbach’s value for EQ-5D-3L was 0.78.

### 2.4. Questionnaire and Scorings

The European Quality of Life 5-Dimension 3-Level (EQ-5D-3L) is a standardized instrument for measuring HRQoL [25,26]. HRQoL is measured in terms of five dimensions (5D): mobility, self-care, usual activities, pain/discomfort, and anxiety/depression. Each dimension has three questions. The respondents self-rate their levels of severity for each dimension using the three levels of the EQ-5D-3L: Score 1: indicating no problem; Score 2: indicating some problems; and Score 3: indicating extreme problems.

### 2.5. Ethical Clearance

Ethical clearance was obtained from the institution’s ethics committee, Grace College of Pharmacy, Palakkad (Approval No: GCP/IEC/112A/2022 dated on 5 July 2022), prior to commencement of this study.

### 2.6. Statistical Analysis

Data were analyzed using IBM SPSS Statistics for Windows, Version 20.0. Categorical variables were expressed as numbers and percentages. The association of sociodemographic factors, types of CAD and lifestyle risk factors, sleep patterns, and dietary habits with HRQoL were analyzed by using multivariate logistic regression analysis. A *p*-value of <0.05 was considered statistically significant.

## 3. Results

This prospective hospital-based study assessed HRQoL and determinants like lifestyle, sleep, and diet in 212 patients with CAD over 9 months. Table 1 represents the sociodemographic and lifestyle factors among CAD patients. The majority of participants, 173 (81.6%), were male, and the minority, 39, were female (18.4%). A large proportion, 177, were aged ≥50 years (83.5%), while 35 (16.5%) were under 50 years. Regarding education, most, 121, had primary education (57.1%), followed by 78 with secondary education or a bachelor’s degree and above (36.8%), and a small number, 13, who were illiterate (6.1%). The majority, 142, were employed (67%), with 70 (33%) being unemployed, retired, or housewives. Most participants, 209, were married (98.6%), and 73 (34.4%) had a family history of CAD. A large number, 151 (71.2%), had comorbid diseases, while 61 (28.8%) did not. Regarding exercise, 129 (60.8%) exercised ≤1 time/week, while 83 (39.2%) exercised ≥2–3 times/week. The majority, 160, had never smoked or were ex-smokers (75.5%) while 52 (24.5%) were current smokers. Alcohol intake was reported by 87 (41.0%); 125 (59.0%) did not consume alcohol. Sleep duration of ≤6 h/night was observed in 143 (67.5%), while 69 (32.5%) slept for >6 h. A larger portion, 151, had moderate-/high-risk dietary habits (71.2%) with 61 (28.8%) having low-risk diets.

Table 2 presents the distribution of EQ-5D-3L questionnaire scores among the study population. Regarding “mobility,” 56 (26.4%) patients reported no problems, 150 (70.8%) had some problems, and 6 (2.8%) were confined to bed. For “self-care,” 156 (73.5%) had no issues, 41 (19.3%) experienced some problems, and 15 (7%) were unable to care for themselves. In terms of “usual activities,” 51 (24%) had no problems, 136 (64.1%) faced some issues, and 25 (11.8%) were unable to perform daily tasks. “Pain/discomfort” was reported by 78 (36.8%), with 134 (63.2%) experiencing no pain, 45 (21.2%) reporting moderate pain, and 33 (15.6%) experiencing extreme pain. Regarding “anxiety/depression,” 28 (13.2%) of CAD patients reported not feeling anxious or depressed, 118 (55.6%) were moderately affected, and 66 (31.1%) experienced extreme anxiety/depression.

Table 3 shows the relationships between sociodemographic and lifestyle factors with EQ-5D-3L domains among CAD patients. Women (89.7%) and patients with comorbid diseases (78.8%) were more likely to report some or extreme problems in the “mobility” dimension (*p* < 0.05). Men (79.2%), those with a family history of CAD (92.7%), illiterate people (92.3%), and people exercising regularly (≥2–3 times/week) were more likely to report some or extreme problems in “usual activities” (*p* < 0.05). Patients with primary education (79.3%), without a family history of CAD (78.4%) or comorbid diseases (77.5%) and who were exercising ≥2–3 times/week were more likely to report no problems with the “self-care” (*p* <0.029) dimension. Patients who exercised ≤1 time/week were more likely to report no pain in the “pain dimension” (*p* < 0.05). Women (97.4%), patients with comorbid diseases (92.7%), and those who exercised ≥2–3 times/week (94%) were more likely to report some or extreme problems in the “anxiety/depression” dimension (*p* < 0.05).

Table 4 presents the multivariate logistic regression analysis evaluating the association between sociodemographic and lifestyle factors with HRQoL among CAD patients. Participants aged ≥50 years (OR = 2.091, 95% CI: 1.042–4.192, *p* = 0.037) and current smokers (OR = 2.045, 95% CI: 1.112–3.978, *p* = 0.049) were significantly associated with “anxiety/depression.” Unemployed, retired, or housewife participants were significantly associated with usual activities (OR = 2.165, 95% CI: 1.098–4.269, *p* = 0.027). Participants with a family history of CAD were significantly associated with “pain/discomfort” (OR = 2.045, 95% CI: 1.034–4.051, *p* = 0.042) and “anxiety/depression” (OR = 2.354, 95% CI: 1.178–4.702, *p* = 0.019). Participants with comorbid diseases were significantly associated with “mobility” (OR = 2.658, 95% CI: 1.221–5.788, *p* = 0.014), “self-care” (OR = 2.743, 95% CI: 1.321–5.698, *p* = 0.012), “usual activities” (OR = 2.512, 95% CI: 1.189–5.312, *p* = 0.019), “pain/discomfort” (OR = 3.021, 95% CI: 1.578–5.932, *p* = 0.004) and “anxiety/depression” (OR = 2.895, 95% CI: 1.478–5.672, *p* = 0.006). A moderate-/high-risk diet was significantly associated with impairments across all EQ-5D-3L domains: “mobility” (OR = 2.341, 95% CI: 1.198–4.571, *p* = 0.012), “self-care” (OR = 2.198, 95% CI: 1.112–4.319, *p* = 0.019), “usual activities” (OR = 2.432, 95% CI: 1.231–4.812, *p* = 0.008), “pain/discomfort” (OR = 2.798, 95% CI: 1.412–5.541, *p* = 0.002), and “anxiety/depression” (OR = 2.978, 95% CI: 1.578–5.772, *p* = 0.001). Factors like gender, educational level, alcohol consumption, sleep duration, and exercise showed no significant associations with HRQoL in CAD patients.

## 4. Discussion

This cross-sectional study aimed to evaluate factors associated with quality of life (QoL) among 212 patients diagnosed with coronary artery disease (CAD) over an 8-month period. In India, CAD has increasingly been recognized as a significant public health concern [27]. The majority of participants in this study were male (81.69%), which aligns with findings from similar studies conducted in Saudi Arabia (79.9% vs. 20.1%) and Iran (68.5% vs. 31.5%) [28,29]. For instance, in a study by Sayad et al., 79.9% of the 498 CAD patients were men, while 20.1% were women [28]. The higher prevalence of CAD among men has been well documented, with the lifetime risk of coronary heart disease (CHD) being 49% for men and 32% for women aged over 40 [30].

This study revealed that CAD was most prevalent in individuals aged over 50, with the mean age of onset in Southeast Asians reported to be 53 [31] as compared with the European average of 63. Hypertension was the most predominant risk factor (76.53%), followed by hyperlipidemia (69.95%) and diabetes mellitus (60.56%), which is consistent with other studies [32]. The relationship between elevated blood pressure and the development of CAD is well understood, as it contributes to the formation of atherosclerotic plaques and worsens coronary perfusion [33].

Regarding lifestyle factors, this study observed low levels of physical activity among patients, with 60.8% reporting ≤1 time/week of exercise. Sedentary behavior is common among CAD patients and includes activities such as watching television, using computers, or working in an office [34,35]. Additionally, 67.4% of patients reported insufficient sleep, with a sleep duration of <6 h per night. Short sleep duration has been associated with subclinical cardiovascular disease and cardiometabolic syndrome, leading to adverse cardiovascular outcomes [36]. These findings emphasize the importance of sleep quality in managing CAD and reducing its impact on QoL [37].

Dietary factors also played a significant role, with 71.2% of participants following a moderate- or high-risk diet. A poor diet is a well-established risk factor for cardiovascular disease and obesity [38]. This study also found that 70.8% of participants reported problems with mobility, 64.1% experienced difficulties with usual activities, and 86.7% experienced moderate to extreme levels of anxiety or depression. This aligns with previous studies that show a high prevalence of mental health issues such as anxiety and depression among CAD patients [39,40], which are significant risk factors for myocardial infarction and mortality [41].

Gender differences were also observed in this study. Female patients and those with comorbid diseases were more likely to report mobility and anxiety/depression issues, suggesting a lower QoL for women compared with men. Previous studies have highlighted the fact that women with CAD report poorer physical functioning and mental health compared with their male counterparts [42,43]. A study involving 8580 patients from 22 European countries, conducted over six months following CHD hospitalization, reported a prevalence of depression ranging from 8.2% to 35.7% in men and 10.3% to 62.5% in women, while anxiety prevalence ranged from 12.0% to 41.8% in men and 21.5% to 63.7% in women [44]. Furthermore, this study found that patients who exercised regularly were more likely to report pain and anxiety/depression, which could be attributed to factors such as intensity of exercise or other lifestyle factors influencing mental health [45,46].

Several sociodemographic and lifestyle factors were found to significantly influence QoL dimensions. For instance, unemployment status, family history of CAD, comorbid diseases, and a moderate- to high-risk diet were significantly associated with physical health issues such as pain and difficulties with usual activities (*p* < 0.05). Similarly, age ≥ 50 years, family history of CAD, smoking, comorbid conditions, and a poor diet were significantly correlated with anxiety and depression (*p* < 0.05). The impact of diet on all dimensions of QoL was also observed, which is consistent with findings from studies in Brazil, Norway, and China [47,48,49] but is different from those in Turkey [50] and Indonesia [51].

Our study underscores the importance of considering both physical and mental health factors in the management of CAD patients. Depression and anxiety have been associated with increased cardiac mortality and higher cardiovascular risk [52]. Therefore, addressing mental health in CAD patients should be a priority in clinical settings to improve survival and QoL [53]. The role of diet is particularly crucial, as a healthy diet has been shown to prevent various cardiovascular risk factors, including dyslipidemia, hypertension, and diabetes, while also providing benefits for mental health [54].

Previous research has consistently demonstrated the negative impact of coronary artery disease (CAD) on health-related quality of life (HRQoL), influenced by sociodemographic, clinical, and psychosocial factors [55]. Similarly, the current study confirms that CAD negatively affects QoL. Specifically, factors such as gender, educational level, alcohol intake, and sleep duration were explored. While these factors showed a non-significant association with all dimensions of QoL (*p* > 0.05), they may still offer valuable insights in future research. Given the findings, it is essential for healthcare providers to evaluate QoL when treating CAD patients. By doing so, providers can tailor their support and treatment strategies to meet individual needs. This study suggests that early evaluation of lifestyle factors, including exercise, diet, and sleep, could be crucial in identifying areas where interventions may be most beneficial. Such evaluations can lead to appropriate referrals and better-targeted support for CAD patients [56].

### Limitations

This study has several limitations. First, as a cross-sectional study, it could only identify variables that may impact the QoL of CAD patients but was unable to establish causal relationships. Therefore, a multi-center longitudinal study is needed to determine the long-term benefits of addressing factors associated with lower QoL. Second, data on treatments received for CAD, cardiac function, and other clinical parameters that may influence QoL were not included in this study. Third, the study sample was limited to 212 patients from a single tertiary hospital in Palakkad, which may not be representative of CAD patients across India. The small sample size has been acknowledged as a major limitation. Eating habits and exercise levels may be specific to this regional population and may differ from national averages. Fourth, selection bias may limit the generalizability of the findings to women, as this study included a greater number of men than women. Fifth, using a disease-specific QoL questionnaire, such as the Seattle Angina Questionnaire or the Hamilton Anxiety Rating Scale, would have provided more detailed insights into CAD patients’ QoL. However, due to practical constraints, we opted for the EQ-5D-3L, which provides a broad measure of health status but may not capture disease-specific nuances.

## 5. Conclusions

This study found that CAD patients undergoing treatment for secondary prevention exhibited inadequate HRQoL. Factors such as being older, a family history of CAD, current smoking, comorbid disease, and a moderate- to high-risk diet were significantly associated with the anxiety/depression dimension. Unemployed status, comorbid disease, family history of CAD and a moderate- to high-risk diet were significantly associated with physical health (problems in usual activities and pain). Moderate to severe anxiety or depression affected 87% of CAD patients. The relationship between anxiety/depression and CAD should be a focus for both physicians and psychiatrists. HRQoL should be regularly monitored and documented as part of treatment strategies and interventions aimed at improving outcomes for CAD patients.

## Figures and Tables

**Table 1:** Sociodemographic and lifestyle factors among CAD patients.

Variables	Number of Patients *n* = 212 (%)
**Gender**	
Male	173 (81.60)
Female	39 (18.39)
**Age group in years**	
<50 years	35 (16.50)
≥50 years	177 (83.49)
**Education**	
Illiterate	13 (6.13)
Primary Education	121 (57.07)
Secondary Education/Bachelor’s Degree and Above	78 (36.79)
**Occupation**	
Unemployed/Retired/Housewife	70 (33.01)
Employed	142 (66.98)
**Marital status**	
Married	209 (98.58)
Unmarried	4 (1.88)
**Family history of CAD**	
Yes (Father/Mother)	73 (34.43)
No	139 (65.56)
**Comorbid disease**	
Yes (HT, DM, Hyperlipidemia, hypothyroidism)	151 (71.22)
No	61 (28.77)
**Exercise**	
≤1 time/week	129 (60.84)
≥2–3 times/week	83 (39.15)
**Smoking**	
Never/Ex-smoker	160 (28.30)
Current smoker	52 (24.52)
**Alcohol intake**	
Yes	87 (41.03)
No	125 (58.96)
**Sleep Duration**	
≤6 h/night	143 (67.45)
>6 h	69 (32.54)
**Dietary habits**	
Low Risk	61 (28.77)
Moderate/High Risk	151 (71.22)

HT: Hypertension, DM: Diabetes Mellitus.

**Table 2:** Distribution based on EQ-5D-3L questionnaire scores among the study population.

Dimensions	Score	Number (%) (*n* = 212)
Mobility	1 (no problems)	56 (26.41)
	2 (some problems)	150 (70.75)
	3 (confined to bed)	6 (2.8)
Self-care	1 (no problems)	156 (73.5)
	2 (some problems)	41 (19.3)
	3 (unable)	15 (7)
Usual activities	1 (no problems)	51 (24)
	2 (some problems)	136 (64.1)
	3 (unable)	25 (11.79)
Pain/Discomfort	1 (no pain)	134 (63.2)
	2 (moderate pain)	45 (21.22)
	3 (extreme pain)	33 (15.56)
Anxiety/Depression	1 (not anxious depressed)	28 (13.2)
	2 (moderately anxious or/depressed)	118 (55.6)
	3 (extreme anxious/depressed)	66 (31.1)

**Table 3:** The relationships between sociodemographic variables and lifestyle factors with EQ-5D-3L domains among CAD patients using the chi-square test.

Parameters	Mobility			Self Care			Usual Activities			Pain			Anxiety/Depression		
	No Problems	Some or Extreme Problems	*p* Value	No Problems	Some or Extreme Problems	*p* Value	No Problems	Some or Extreme Problems	*p* Value	No Problems	Some or Extreme Problems	*p* Value	No Problems	Some or Extreme Problems	*p* Value
**Gender**															
Male	51 (29.5)	122 (70.5)	0.013 *	124 (71.6)	49 (28.3)	0.300	36 (20.8)	137 (79.2)	0.044 *	113 (65.6)	60 (34.7)	0.101	27 (15.6)	146 (84.4)	0.029 *
Female	4 (10.3)	35 (89.7)		31 (79.4)	08 (25.5)		14 (35.9)	25 (64.1)		20 (51.3)	19 (48.7)		01 (2.6)	38 (97.4)	
**Age group**															
<50 years	9 (25.7)	26 (74.3)	0.973	29 (82.2)	06 (17.1)	0.173	11 (31.4)	24 (68.6)	0.264	22 (62.2)	13 (37.2)	0.962	03 (8.8)	32 (91.2)	0.375
>50 years	46 (26)	131 (74)		127 (71.8)	50 (28.2)		40 (22.6)	137 (77.4)		112 (63.3)	65 (36.7)		25 (14.1)	152 (85.9)	
**Education**															
Illiterate	6 (46.1)	07 (53.8)	0.149	06 (46.1)	07 (53.8)	0.016 *	01 (7.7)	12 (92.3)	0.005 *	08 (61.5)	05 (38.5)	0.727	03 (33.1)	10 (76.9)	0.496
Primary	27 (22.3)	94 (77.7)		96 (79.3)	25 (20.6)		41 (33.9)	80 (66.1)		74 (61.2)	47 (38.8)		14 (11.6)	107 (88.4)	
Secondary/ Degree	22 (28.2)	56 (71.8)		53 (67.9)	25 (32.1)		09 (11.5)	69 (88.5)		52 (66.7)	26 (33.3)		10 (12.8)	68 (87.2)	
**Employment**															
Unemployed/ Retired	14 (20)	56 (80)	0.136	52 (74.3)	18 (25.7)	0.870	12 (17.1)	58 (82.9)	0.098	45 (64.3)	25 (35.7)	0.819	07 (10)	63 (90)	0.332
Employed	42 (29.6)	100 (70.4)		104 (73.2)	38 (26.8)		39 (27.5)	103 (72.5)		89 (62.8)	53 (37.3)		21 (14.9)	121 (85.2)	
**F/H Of CAD**															
Yes	22 (30.1)	51 (69.9)	0.373	47 (64.9)	26 (35.6)	0.027 *	11 (15.1)	62 (84.9)	0.026 *	44 (60.3)	29 (39.7)	0.520	14 (19.2)	59 (80.8)	0.062
No	34 (24.5)	105 (75.5)		109 (78.4)			40 (28.8)	99 (71.2)		90 (64.7)	49 (35.3)		14 (10.1)	125 (89.9)	
**Comorbid disease**															
Yes	32 (21.2)	119 (78.8)	0.006 *	117 (77.5)	34 (22.5)	0.042 *	41 (27)	110 (72.8)	0.097	93 (61.6)	58 (38.4)	0.442	11 (7.3)	140 (92.7)	0.0006 *
No	24 (39.3)	37 (60.7)		39 (63.9)	22 (36)		10 (16.4)	51 (83.6)		41 (67.2)	20 (32.8)		17 (27.9)	44 (72.1)	
**Exercise**															
≤1 times/week	34 (26.4)	95 (73.6)	0.980	88 (68.2)	41 (31.8)	0.027 *	23 (17.8)	106 (82.2)	0.008 *	89 (69)	40 (31)	0.029 *	23 (17.8)	106 (82.2)	0.013 *
≥2–3 times/week	22 (26.5)	61 (73.5)		68 (82)	15 (18.1)		28 (33.7)	55 (66.3)		45 (54.2)	38 (45.8)		05 (06)	78 (94)	
**Smoking**															
Never/ Ex-smoker	44 (27.5)	116 (72.5)	0.529	118 (73.8)	42 (26.3)	0.923	35 (21.9)	125 (78.1)	0.192	102 (63.8)	58 (36.2)	0.773	22 (13.8)	138 (86.2)	0.682
Current	12 (23.1)	40 (76.9)		38 (73)	14 (26.9)		16 (30.8)	36 (69.2)		32 (61.5)	20 (38,5)		06 (11.5)	46 (88.5)	
**Alcohol intake**															
Yes	38 (30.4)	87 (69.6)	0.114	94 (75.2)	31 (24.8)	0.522	24 (19.2)	101 (80.8)	0.0714	81 (64.8)	44 (35.2)	0.564	18 (14.4)	107 (85.6)	0.538
No	18 (20.7)	69 (79.3)		62 (71.3)	25 (28.7)		26 (29.9)	61 (70.1)		53 (60.9)	34 (39.1)		10 (11.5)	77 (88.5)	
**Sleep duration**															
≤6 h/night	40 (28)	103 (72)	0.459	101 (70.6)	42 (29.4)	0.159	31 (21.7)	112 (78.3)	0.243	91 (63.6)	52 (36.4)	0.852	21 (14.7)	122 (85.3)	0.360
>6 h/night	16 (23.2)	53 (76.8)		55 (79.7)	14 (20.3)		20 (29)	49 (71)		43 (62.3)	26 (37.7)		07 (10.1)	62 (89.9)	
**Diet**															
Low risk	20 (32.8)	41 (67.2)	0.181	43 (70.5)	18 (29.5)	0.516	14 (23)	47 (77)	0.810	34 (55.7)	27 (44.3)	0.151	11 (18)	50 (82)	0.187
Moderate/ High risk	36 (23.8)	115 (76.2)		113 (74.8)	38 (25.2)		37 (24.5)	114 (75.5)		100 (66.2)	51 (33.8)		17 (11.3)	134 (88.7)	

*p* < 0.05 * significance.

**Table 4:** The multivariate logistic regression analysis of coronary artery disease patients in association with sociodemographic variables and lifestyle factors versus the EQ-5D-3L domains.

Parameters	Mobility			Self Care			Usual Activities			Pain			Anxiety/Depression		
	OR Ratio	CI	*p* Value	OR Ratio	CI	*p* Value	OR Ratio	CI	*p* Value	OR Ratio	CI	*p* Value	OR Ratio	CI	*p* Value
**Gender**															
Male	Reference														
Female	0.108	(0.00065, 1.797)	0.394	1.102	(0.541, 2.246)	0.792	1.432	(0.712, 2.882)	0.321	0.876	(0.432, 1.774)	0.712	1.654	(0.843, 3.241)	0.178
**Age group**															
<50 years	Reference														
≥50 years	1.512	(0.789, 2.897)	0.204	1.873	(0.942, 3.721)	0.073	1.326	(0.678, 2.592)	0.412	1.765	(0.879, 3.512)	0.092	2.091	(1.042, 4.192)	0.037 *
**Education**															
Illiterate	0.876	(0.432, 1.774)	0.712	1.142	(0.578, 2.256)	0.693	1.321	(0.641, 2.723)	0.415	0.897	(0.451, 1.783)	0.782	1.621	(0.789, 3.012)	0.213
Primary	0.763	(0.382, 1.523)	0.444	0.932	(0.481, 1.805)	0.832	1.012	(0.521, 1.967)	0.973	1.023	(0.542, 1.932)	0.951	1.154	(0.578, 2.212)	0.693
Secondary/Degree	Reference														
**Employment**															
Unemployed/Retired/Housewife	1.932	(0.921, 4.052)	0.082	1.982	(0.973, 4.034)	0.059	2.165	(1.098,4.269)	0.027 *	1.765	(0.879, 3.512)	0.092	1.654	(0.843, 3.241)	0.178
Employed	Reference														
**Family history of CAD**															
Yes	1.421	(0.739, 2.731)	0.289	1.543	(0.812, 2.932)	0.201	1.782	(0.893, 3.456)	0.087	2.045	(1.034, 4.051)	0.042 *	2.354	(1.178, 4.702)	0.019 *
No	Reference														
**Comorbid disease**															
Yes	2.658	(1.221, 5.788)	0.014 *	2.743	(1.321, 5.698)	0.012 *	2.512	(1.189, 5.312)	0.019 *	3.021	(1.578, 5.932)	0.004 *	2.895	(1.478, 5.672)	0.006 *
No	Reference														
**Exercise**															
≤1 time/week	0.743	(0.389, 1.419)	0.371	0.821	(0.412, 1.632)	0.482	0.765	(0.389, 1.512)	0.404	0.892	(0.431, 1.732)	0.563	0.698	(0.321, 1.432)	0.275
≥2–3 times/week	Reference														
**Smoking**															
Never/Ex-smoker	Reference														
Current	1.567	(0.812, 3.023)	0.182	1.642	(0.821, 3.278)	0.193	1.732	(0.872, 3.456)	0.221	1.856	(0.912, 3.672)	0.174	2.045	(1.112, 3.978)	0.049 *
**Alcohol intake**															
Yes	0.984	(0.513, 1.888)	0.961	1.021	(0.542, 1.932)	0.897	0.978	(0.498, 1.892)	0.932	1.145	(0.598, 2.231)	0.674	1.298	(0.678, 2.489)	0.38
No	Reference														
**Sleep duration**															
≤6 h/night	1.276	(0.649, 2.507)	0.483	1.398	(0.721, 2.721)	0.291	1.321	(0.678, 2.562)	0.402	1.523	(0.789, 2.931)	0.196	1.674	(0.843, 3.241)	0.178
> 6 h/night	Reference														
**Diet**															
Low Risk	Reference														
Moderate/High Risk	2.341	(1.198, 4.571)	0.012	2.198	(1.112, 4.319)	0.019	2.432	(1.231, 4.812)	0.008	2.798	(1.412, 5.541)	0.002	2.978	(1.578, 5.772)	0.001

*p* < 0.05 * significance; CI: Confidence Interval, OR-Odds ratio.

## Data Availability

The original contributions presented in this study are included in the article; further inquiries can be directed to the corresponding author.
